# Supplementary material for: Contextual constraints and dilemmas influencing health providers’ prescription practices in a conflict-affected area: Qualitative insights from Mopti, Mali
Source: PLOS Glob Public Health. 2025 Oct 16;5(10):e0004621. doi: 10.1371/journal.pgph.0004621 (PMC12530568; doi:10.1371/journal.pgph.0004621)
Supplement: Attachment — Submitted filename: Responses (1).docx [file pgph.0004621.s002.docx]

| **Manuscript** ID:[PGPH-D-25-00971] - Titled **" Contextual constraints and dilemmas influencing health providers’ prescription practices in a conflict-affected area: qualitative insights from Mopti, Mali**" | |
| --- | --- |
| **Editor or Reviewer's comments** | **Author's responses** |
| **Editor:** |  |
| 1. **Language, Grammar, and Writing Style:** The manuscript requires thorough proofreading to correct a range of grammatical and stylistic issues. I observed multiple instances of awkward phrasing, overly long sentences, inconsistent use of abbreviations, and problems with cohesion. The clarity and impact of the paper would greatly benefit from breaking down complex sentences and ensuring correct grammar throughout | Thank you for pointing out. The article has been thoroughly proofread and revised by two native English-speaking co-authors. Sentences have been simplified, shortened where necessary, and syntax and style problems corrected to improve the fluidity and clarity of the text. Abbreviations have been completely revised. All the abbreviations used in the manuscript were defined when they first appeared in the text. Furthermore, in accordance with the journal's recommendations, only necessary abbreviations have been retained, and those used less than three times have been entirely deleted or rewritten to avoid any confusion. |
| 2. **Title Accuracy:** The current title refers to “conflict-affected areas” (plural), which is misleading, as the study focuses solely on Mopti, Mali. Please consider revising the title to accurately reflect the geographic scope of the research. | Thank you for your pertinent comment concerning the geographical scope mentioned in the title. This comment has been considered. It should be emphasized that this is not a generalization, but an analysis focusing on the specific case of the Mopti region in Mali. The title has been adjusted accordingly to better reflect the actual scope of the study. |
| 3. **Overlooked Dimension: Financial or Corporate Incentives:** A major limitation of the current analysis is the absence of discussion around financial or corporate interests—particularly the influence of pharmaceutical companies. There is substantial global literature linking pharmaceutical incentivization to inappropriate prescribing practices (e.g., polypharmacy). It would be important to address whether this issue arose in your data. Did any participants mention pharmaceutical influence, or how companies might operate in or exploit conflict settings? If this theme did not emerge, it would be helpful to acknowledge its absence and reflect on why that may be the case. | Thank you for this pertinent comment, which has enriched our analysis. In response, we have added a specific section to the discussion to look in more detail at the issue of financial incentives and industry interests, including the potential role of pharmaceutical companies in inappropriate prescribing practices, including in contexts of conflict." |
| 4. **Narrative Framing of Results:** While the structure of the results section is appropriate, it is currently too reliant on quoted material with limited analytical interpretation. For an international audience, it is essential that you provide a clearer narrative voice to guide readers through the themes. Rather than long descriptive passages, I recommend integrating brief but thoughtful reflections that help contextualize and interpret the quotes. This will strengthen the coherence and analytical depth of the results. | Thank you for your pertinent comment concerning the narrative construction of the results section. Following your comment, we have reworked the entire section to improve its clarity and analytical coherence. More specifically, the verbatims have been shortened to lighten the text and avoid descriptive overload. This has allowed us to include more succinct but focused analytical reflections, designed to contextualise the quotations and highlight the main lessons from each theme. We have also been careful to assert a clearer narrative voice to guide the reader through the findings, particularly from an international reading perspective. |
| **Reviewer #1 :** |  |
| Well written manuscript. Appreciate the flowless translation from French. Excellent review of literature. Abbreviations, such as IPM, NGO could be defined at first mention for ease of reading | We would like to thank you for your positive comments regarding the quality of the writing, the translation and the literature review. About abbreviations, we followed an approach aimed at defining those that were not mentioned more than three times, each occurrence. This decision was taken to ensure the clarity of the text for readers, while avoiding explanatory overload for infrequent abbreviations. |
| A couple of questions: Lines 152 Reference? | Thank you for this clarification. Line 152 does refer to citation no. 21: Ag Ahmed, M. A., Maiga, A., Coulibaly, I., Kielmann, K., & Ravinetto, R. Irrational medicine use and its associated factors in conflict-affected areas in Mali: A cross-sectional study. Global Health. 2023. |
| o Lines 156 NGO crucial role in what? | The private sector and NGOs also play a crucial role in healthcare provision. |
| o Lines 207 Quantitative survey? | Thank you for your question. An interview guide was indeed developed based on the existing literature, as well as our previous experience in conducting a quantitative survey on irrational prescribing. However, the data presented in the manuscript come exclusively from the qualitative component of the study. |
| o Lines 277 The sentence is not clear ? | Thank you for your comment. The sentence mentioned has been reworded to improve its flow and make it clearer and more understandable for the reader |
| o Lines 333, 341 I see empty [...]. Not sure if this is intentional | Thank you for your comment. The empty square brackets [...] were intentionally used to indicate passages in the verbatims that were deemed too long, repetitive or irrelevant for the analysis. They were used to shorten quotations while preserving the meaning and coherence of the comments, in accordance with good practice in the presentation of qualitative data. The aim of this approach was to improve readability without altering the essential content of the statements |
| o Lines 598 To improve working conditions aside staff, training, supervision, and monitoring, you may want to ensure security. | Thank you for your comment. The relevant section has been revised to remove any ambiguity and to clarify the importance of safety as an essential element in improving working conditions, in addition to personnel, training, supervision and monitoring |
